# The association between total bilirubin and serum triglyceride in both sexes in Chinese

**DOI:** 10.1186/s12944-018-0857-7

**Published:** 2018-09-12

**Authors:** Xuemei Zhang, Zhaowei Meng, Xue Li, Ming Liu, Xiaojun Ren, Mei Zhu, Qing He, Qing Zhang, Kun Song, Qiyu Jia, Chunmei Zhang, Xiaoran Wang, Xiangxiang Liu

**Affiliations:** 1 Department of Nuclear Medicine, Tianjin Medical University General Hospital, Anshan Road No. 154, Heping District, Tianjin, 300052 P.R China; 2 Department of Endocrinology and Metabolism, Tianjin Medical University General Hospital, Tianjin, P.R China; 3 Department of Health Management, Tianjin Medical University General Hospital, Tianjin, P.R China

**Keywords:** Total bilirubin (TB), Triglyceride (TG), Gender, Age

## Abstract

**Objective:**

Dyslipidemia can cause some chronic diseases. Bilirubin is reported to have anti-inflammatory and anti-oxidant properties. We aimed to explore the relationship between triglyceride and total bilirubin (TB) in a large Chinese cohort.

**Methods:**

A total of 80,298 healthy Chinese (48,971 male, 31,327 female) enrolled in this cross-sectional study. Clinical data were collected from participants. Database was divided according to sex and age. The relationship between triglyceride and TB was analyzed by quartiles of TB. Levels of triglyceride were compared in different TB subgroups by one-way analysis of variance and independent sample’s t-test. Crude and adjusted odds ratios of triglyceride for TB with 95% confidence intervals were analyzed by binary logistic regression models.

**Results:**

Overall, men showed higher levels of TB and triglyceride than women. In people older than 60 years, women had higher triglyceride levels than men. Linear logistic regression analyses showed a negative relationship between triglyceride and TB in both genders. Men showed significantly higher overall incidence of high triglyceride than women. Men less than 60 years old showed a higher incidence of high triglyceride. For women older than 60 years, the incidence of high triglyceride was higher. However, high TB showed no protective effects on both genders from high TG in the binary logistic regression models. TB showed a detrimental effect on high TG in model 2 which included TB as a covariate. But that effect disappeared after other confounding factors were included.

**Conclusions:**

Our findings showed no association between TB and triglyceride in Chinese.

## Background

Bilirubin is the end product of heme catabolism [1]. In the past, TB was considered to be a toxic waste [2]. However, recent studies have shown that TB had protective properties against various diseases. For instance, vasodilatation, anti-oxidation, anti-inflammatory, anti-mutation, immune regulation, anti-proliferation and anti-apoptosis of vascular cells had been identified [3, 4]. After a comprehensive literature review, a number of studies investigated the relationship between TB and diseases like cardiovascular disease [5–9], metabolism syndrome (MS) [10–13], Gilbert’s syndrome (GS) [14, 15], diabetes mellitus [16, 17], inflammation [18], Williams–Beuren syndrome [19]. There were no paper investigating TB and triglyceride (TG) directly, several papers mentioned that there were a negative relationship between them though. But these papers had limitations, for instance: the age distribution of the subjects was uneven [11, 12, 20, 21], gender differences were not studied [11, 13], small recruitment size [9, 21], limited studies in Chinese people [5].

Based on the existence of the above problems, the main purpose of this study is to further clarify the relationship between TB and TG and the gender and age differences.

## Methods

### Design and recruitment

We had a multidisciplinary team in General Hospital Affiliated to Tianjin Medical University, carrying out a survey for nearly 10 years. The protocol of the community-based health survey was reported in our previous articles [22–29]. In simple terms, the questionnaires were distributed to the participants who reported to be healthy ostensibly. After an overnight fast (time ≥ 8 h), blood samples were obtained to measure levels of blood parameters immediately. In order to control confounding factors, the following situations were excluded: participants who had histories of blood, liver, kidney, stomach, thyroid inflammation, infection, cancer, and autoimmune diseases; subjects taking any medication which might affect TB and TG; subjects with TB of 40 μmol/L or higher, alanine aminotransferase (ALT) of 100 U/L or higher; excessive drinking; pregnancy. To avoid the increase of TB caused by hemolysis, samples after centrifugation were visually tested for hemolysis. For the purpose of this particular study, the data was collected from September 2010 to September 2015. A total of 80,298 participants (48,971 males and 31,327 females) met the requirements and had sufficient data for our analysis.

### Measurements

All participants underwent anthropometric measurements and fasting blood glucose tests during their visitation to our institution. They were instructed to wear light indoor clothing and take off their shoes to measure body height (BH) and body weight (BW). Body mass index (BMI) was calculated by dividing BW (kilograms) by the square of BH (meters^2^). Systolic blood pressure (SBP) and diastolic blood pressure (DBP) were determined with a sphygmomanometer. Biochemical indicators, including: ALT, TB, blood urea nitrogen (BUN), creatinine (Cr), uric acid (UA), TG, total cholesterol (TC), high-density lipoprotein cholesterol (HDL), low-density lipoprotein cholesterol (LDL), glucose (G) were obtained by an auto-analyzer (Hitachi Corporation, Tokyo, Japan).

The laboratory reference standard of parameters were as follows: ALT 5–40 U/L; TB 3.4–20 μmol/L; BUN 1.7–8.3 mmol/L; Cr 44–115 μmol/L; TG 0.57–1.71 mmol/L; TC 3.59–5.17 mmol/L; HDL 0.8–2.2 mmol/L; LDL 1.33–3.36 mmol/L; G 3.6–5.8 mmol/L.

High TG was defined as TG ≥ 1.70 mmol/l. Participants were divided into three groups according to age (first group: age ≤ 30; second group: 30 < age ≤ 60; third group: age > 60).

### Statistical analysis

Data were expressed as mean ± standard deviation (SD). All analyses were performed separately by gender and age groups. The difference of each index was measured by using the independent sample’s t test. Pearson bivariate correlation was performed among TB and other variables. Linear Logistic regression was used to evaluate the independent relationship between TG and TB and adjust for possible confounding factors, including age, BMI, SBP, DBP, BUN, UA, Cr, ALT, TC, LDL, HDL and G. The concentration of TB was divided into four quartiles. Inter-group prevalence differences of high TG were compared by using Chi square test. Binary logistic regression models were performed to analyze crude and adjusted odds ratios for high TG with 95% confidence intervals. Our statistics were achieved by software of Statistical Package for Social Sciences (SPSS version 17.0, Chicago, IL, USA). Significance was defined as *P* < 0.05.

## Results

### Characteristics of the participants in different genders

There were differences among the parameters with respect to opposite gender (Table 1). Females were older than males. The levels of TC, HDL and LDL are higher in females than in males. On the contrary, all the other parameters were significantly higher in males than in females. Differences also existed among the data with respect to the three age groups (Table 2). At the age of less than 30 years, all parameters except HDL were lower in women than men. Between ages 30 and 60, both TC and HDL levels were higher for women than men. At ages over than 60 years, women had higher levels of SBP, TC, HDL, and LDL than men. There was no significant difference in the level of TG at this age. In men, the levels of SBP, DBP, BUN, TC, LDL, and G increased with age. The levels of BMI, ALT, and TG first increased with age and then decreased, whereas TB, Cr, and HDL reversed. Only UA gradually declined with age. In women, the levels of BMI, SBP, DBP, ALT, BUN, Cr, UA, TC, LDL, and G increased with age. TB and TG first increased with age and then decreased. Only HDL gradually declined with age.

**Table 1:** Participant characteristics

Parameters	Males	Females	T value
Case number	48,971	31,327	
Age (years)	46.57 ± 12.19	47.72 ± 13.13	−12.661**
BMI (kg/m2)	25.91 ± 3.22	24.00 ± 3.48	79.585**
SBP (mmHg)	125.41 ± 16.05	121.35 ± 18.34	33.038**
DBP (mmHg)	81.04 ± 11.08	74.82 ± 10.26	79.736**
ALT (U/L)	27.02 ± 14.85	18.51 ± 10.70	87.929**
TB (μmol/L)	13.69 ± 5.44	11.23 ± 4.53	66.494**
BUN (mmol/L)	5.01 ± 1.24	4.43 ± 1.22	65.614**
Cr (μmol/L)	79.29 ± 11.34	59.84 ± 9.58	251.494**
UA (μmol/L)	362.23 ± 74.75	264.72 ± 59.84	194.452**
TG (mmol/L)	1.81 ± 1.32	1.28 ± 0.87	62.513**
TC (mmol/L)	5.13 ± 0.95	5.23 ± 1.03	−15.311**
HDL (mmol/L)	1.28 ± 0.31	1.56 ± 0.36	− 117.095**
LDL (mmol/L)	3.09 ± 0.83	3.11 ± 0.91	−2.518**
G (mmol/L)	3.34 ± 0.93	3.12 ± 0.91	31.524**

*BMI* Body mass index, *SBP* Systolic blood pressure, *DBP* Diastolic blood pressure, *ALT* Alanine aminotransferase, *TB* Total bilirubin, *BUN* Blood urea nitrogen, *Cr* Creatinine, *UA* Uric acid, *TG* Triglycerides, *TC* Total cholesterol, *HDL* High-density lipoprotein, *LDL* Low-density lipoprotein, *G* Glucose

**P < 0.01 (analyzed by independent sample’s t test)

**Table 2:** Participant characteristics of different age groups

Parameters	Age ≤ 30			30 < age ≤ 60			Age > 60		
sex	male	female	F values	male	female	F values	male	female	F values
Case number	5257	3609		37,755	22,527		5959	5191	
BMI (kg/m2)	24.88 ± 3.76	21.46 ± 3.02	190.684**	26.07 ± 3.14	24.02 ± 3.26	50.452**	25.78 ± 3.01	25.66 ± 3.66	173.250**
SBP (mmHg)	119.65 ± 11.83	109.77 ± 10.83	28.373**	124.33 ± 15.29	118.97 ± 16.24	119.107**	137.29 ± 18.30	139.72 ± 18.65	3.936*
DBP (mmHg)	75.11 ± 9.01	69.38 ± 7.91	57.447**	81.61 ± 11.16	74.79 ± 10.24	155.736**	82.66 ± 10.64	78.76 ± 10.07	14.165**
ALT (U/L)	27.68 ± 16.89	14.71 ± 8.45	1303.019**	27.82 ± 14.94	18.79 ± 10.99	2083.180**	21.43 ± 10.55	19.94 ± 10.21	6.966**
TB (μmol/L)	14.24 ± 5.95	11.79 ± 5.22	60.727**	13.60 ± 5.41	11.09 ± 4.49	707.774**	13.80 ± 5.10	11.49 ± 4.15	181.755**
BUN (mmol/L)	4.76 ± 1.15	3.95 ± 1.05	26.320**	4.98 ± 1.20	4.35 ± 1.16	27.649**	5.43 ± 1.38	5.08 ± 1.31	9.701**
Cr (μmol/L)	79.27 ± 10.81	58.19 ± 8.89	125.779**	78.97 ± 11.03	59.29 ± 8.90	875.220**	81.32 ± 13.36	63.37 ± 11.81	82.126**
UA (μmol/L)	368.08 ± 75.42	258.21 ± 53.95	312.995**	363.60 ± 74.22	259.99 ± 57.42	1354.176**	348.40 ± 75.92	289.78 ± 67.26	61.480**
TG (mmol/L)	1.39 ± 1.03	0.85 ± 0.50	508.804**	1.91 ± 1.40	1.27 ± 0.88	1688.890**	1.58 ± 0.95	1.64 ± 0.89	0.624
TC (mmol/L)	4.71 ± 0.87	4.61 ± 0.82	20.302**	5.17 ± 0.94	5.21 ± 1.00	84.977**	5.19 ± 0.94	5.78 ± 1.04	41.255**
HDL (mmol/L)	1.30 ± 0.30	1.62 ± 0.35	125.824**	1.28 ± 0.31	1.56 ± 0.36	803.426**	1.31 ± 0.32	1.53 ± 0.36	91.632**
LDL (mmol/L)	2.81 ± 0.76	2.61 ± 0.73	17.376**	3.11 ± 0.84	3.09 ± 0.88	58.622**	3.19 ± 0.83	3.53 ± 0.93	50.946**
G (mmol/L)	4.82 ± 0.57	4.67 ± 0.48	16.010**	5.36 ± 1.28	5.02 ± 0.83	1047.438**	5.73 ± 1.50	5.55 ± 1.36	35.448**

*BMI* Body mass index, *SBP* Systolic blood pressure, *DBP* Diastolic blood pressure, *ALT* Alanine aminotransferase, *TB* Total bilirubin, *BUN* Blood urea nitrogen, *Cr* Creatinine, *UA* Uric acid, *TG* Triglycerides, *TC* Total cholesterol, *HDL* High-density lipoprotein, *LDL* Low-density lipoprotein, *G* Glucose

**P < 0.01 (analyzed by independent sample’s t test)

### Correlations between TG and other key variables

TG demonstrated significant positive relationships with most of the other variables, including age, BMI, SBP, DBP, ALT, BUN, UA, TC, LDL and G in men, as well as age, BMI, SBP, DBP, ALT, BUN, UA, TC, LDL, Cr and G in women. TB and HDL in both genders and Cr in males had significant negative relationships with TG (Table 3).

**Table 3:** Pearson bivariate correlations between TG and other variables in different genders

Parameters	Correlation coefficients for males	Correlation coefficients for females
Age	0.027**	0.311**
BMI	0.244**	0.320**
SBP	0.116**	0.298**
DBP	0.171**	0.245**
ALT	0.245**	0.225**
BUN	0.009*	0.078**
Cr	−0.008	0.073**
UA	0.238**	0.287**
TB	−0.099**	−0.073**
TC	0.314**	0.351**
HDL	−0.316**	−0.393**
LDL	0.122**	0.249**
G	0.185**	0.245**

*BMI* Body mass index, *SBP* Systolic blood pressure, *DBP* Diastolic blood pressure, *ALT* Alanine aminotransferase, *TB* Total bilirubin, *BUN* Blood urea nitrogen, *Cr* Creatinine, *UA* Uric acid, *TG* Triglycerides, *TC* Total cholesterol, *HDL* High-density lipoprotein, *LDL* Low-density lipoprotein, *G* Glucose

* *P* < 0.05, ** P < 0.01

### Relationship between TG and TB by logistic regression analyses

Linear logistic regression analyses were conducted and negative relationship between TG and TB could be rendered in the following equations. For male: TG = 0.004 × DBP** - 0.002 × age** - 0.005 × BMI** - 0.002 × SBP** - 0.008 × TB** + 2.210 × TC** - 2.453 × HDL** - 2.076 × LDL** + 0.077 × G** + 0.003 × ALT** + 0.005 × BUN - 0.003 × Cr** + 0.001 × UA** - 0.326** (TB R^2^ = 55.6%). For female: TG = 0.033 × G** + 0.000 × age* + 0.000 × SBP + 0.000 × DBP - 0.003 × BMI** - 0.002 × TB** - 0.003 × BUN + 2.239 × TC** - 2.340 × HDL** - 2.208 × LDL** + 0.001 × ALT* + 0.000 × Cr + 0.000 × UA** - 0.011 (TB R^2^ = 74.9%).

### Incidence of high TG according to TB quartiles

**Fig. 1:**
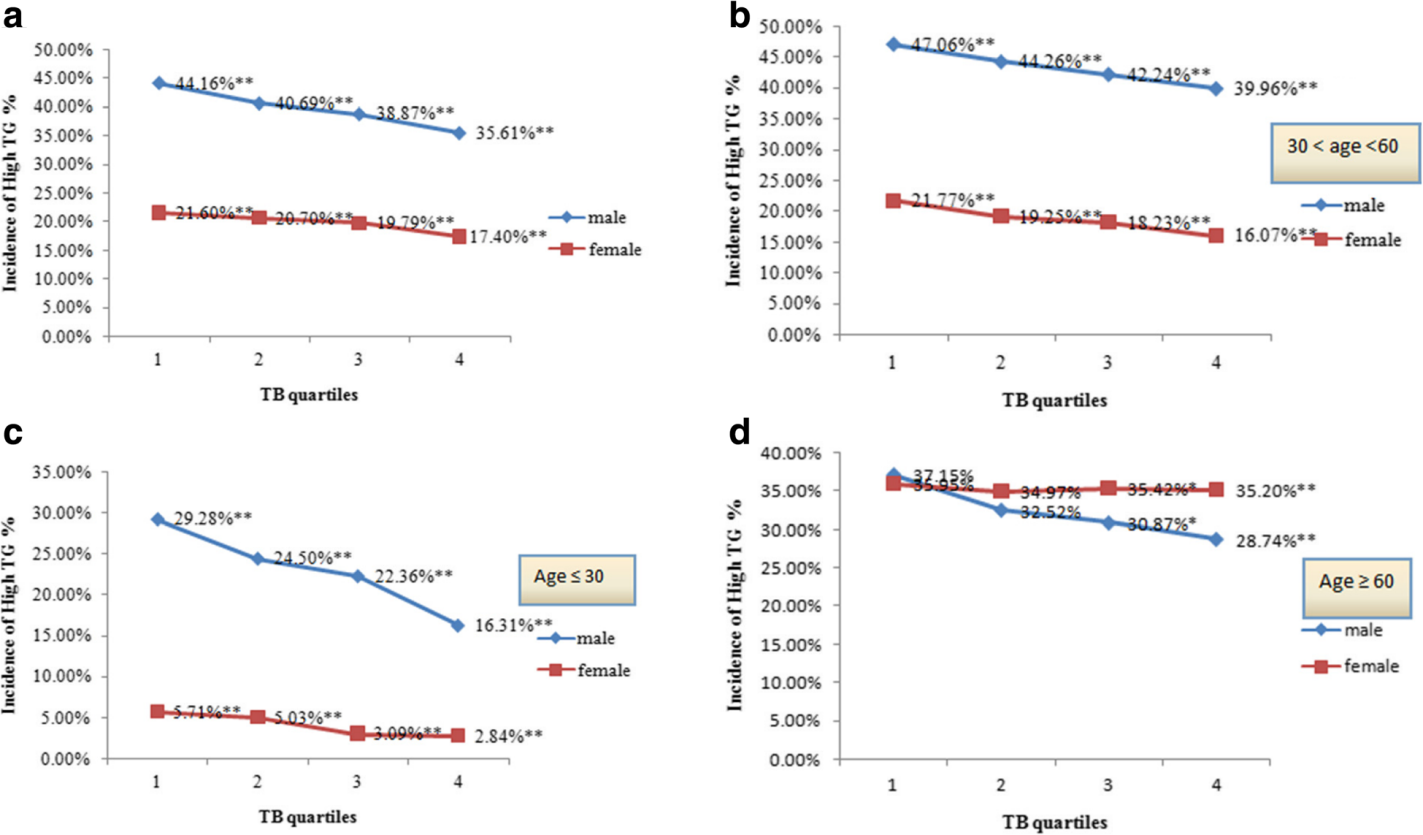
Incidence of high TG in different genders by TB quartiles. **a**: prevalence of all participants (male: TB ≤ 9.9 umol/L, 9.9 umol/L < TB ≤ 12.8 umol/L, 12.8 umol/L < TB ≤ 16.6 umol/L, TB ≥ 16.6 umol/L; female: TB ≤ 8.1 umol/L, 8.1 umol/L < TB ≤ 10.4 umol/L, 10.4 umol/L < TB ≤ 13.5 umol/L, TB ≥ 13.5 umol/L). The prevalence of participants in different age groups was also analyzed separately. (**b**: age ≤ 30, **c**: 30 < age ≤ 60, **d**: age > 60). * *P* < 0.05, ** *P* < 0.01

First, TB quartiles were calculated and incidence of high TG was compared between genders. Males showed significantly higher overall incidence of high TG than females. Detailed incidences in TB quartiles also demonstrated the same pattern of differences between the opposite sexes. The incidence decreased in higher TB quartiles in both genders (Fig. 1a). The incidence of high TG was compared between men and women of different age groups. Men younger than 60 years old had a higher incidence of high TG. For post-menopausal women aged more than 60, the incidence was higher. The incidence of high TG in men decreased with increasing TB quartiles, and this trend was only observed in women under 60 years of age (Fig. 1b, c and d).

### Risks of high TG in different genders

Binary logistic regression models were implemented to calculate the risks of high TG in different genders (Table 4, Table 5). In model 1, TB quartiles were designated as the categorical variable, with the lowest quartile as the reference. Model 1 included TB quartiles, age, BMI, SBP, DBP, ALT, BUN, Cr, UA, TC, HDL, LDL and G as covariates, Model 2 and 3 analyzed TB as a continuous variable. Model 2 included TB as a covariate and model 3 included TB, age, BMI, SBP, DBP, ALT, BUN, Cr, UA, TC, HDL, LDL and G as covariates. All participants were analyzed in different genders (Table 4). In model 2, TB showed a detrimental effect on high TG both in men and women; for instance, the adjusted odd ratio (OR) of TB showed such a role (males: 0.976; females: 0.974). However, this effect disappeared in other models when we added age, BMI, SBP, DBP, ALT, BUN, Cr, UA, TC, HDL, LDL and G as covariates. All the other ORs were not significant. In addition, there was no difference in the result of binary logistic regression analysis based on different age groups (Table 5). Therefore, the current study did not indicate that TB could be used as a risk factor for predicting high TG in both genders.

**Table 4:** The risks of high TG according to TB quartiles in different genders

TB Quartiles	Males		Females	
Model 1	TB values	Adjusted OR (CI)^	TB values	Adjusted OR (CI)^
TB Quartile 1	TB < 9.90 (μmol/L, reference)		TB < 8.10 (μmol/L, reference)	
TB Quartile 2	9.90 ≤ TB < 12.80	0.931 (0.832–1.041)	8.10 ≤ TB < 10.40	0.948 (0.774–1.161)
TB Quartile 3	12.80 ≤ TB < 16.60	0.924 (0.825–1.036)	10.40 ≤ TB < 13.50	0.857 (0.698–1.054)
TB Quartile 4	TB ≥ 16.60	0.924 (0.823–1.038)	TB ≥ 13.50	0.935 (0.752–1.163)
Model 2		0.976 (0.972–0.979)**		0.974 (0.968–0.980)**
Model 3		0.996 (0.989–1.004)		0.991 (0.973–1.009)

*TB* Total bilirubin, *OR* Odds ratio, *CI* Confidence interval

^Logistic regression model 1 with TB Quartile 1 as reference, including age, body mass index, blood pressure, alanine aminotransferase, blood urea nitrogen, creatinine, uric acid, total cholesterol, high-density lipoprotein, low-density lipoprotein and glucose as covariates

Model 2 and 3 analyzed TB as a continuous variable. Model 2 included TB as a covariate and model 3 included age, body mass index, blood pressure, alanine aminotransferase, blood urea nitrogen, creatinine, uric acid, total cholesterol, high-density lipoprotein, low-density lipoprotein and glucose as covariates

* *P* < 0.05, ** *P* < 0.01

**Table 5:** The risks of high TG according to TB quartiles among people in different age groups

TB quartile	Male		Female	
	TB values	Adjusted OR (CI)^	TB values	Adjusted OR (CI)^
Age ≤ 30 Model 1				
TB Quartile 1	TB < 10.0 (μmol/L, reference)		TB < 8.10 (μmol/L, reference)	
TB Quartile 2	10.00 ≤ TB < 13.10	0.947 (0.847–1.060)	8.10 ≤ TB < 10.80	2.047 (0.748–5.604)
TB Quartile 3	13.10 ≤ TB < 17.40	0.948 (0.846–1.062)	10.80 ≤ TB < 14.40	1.072 (0.392–2.928)
TB Quartile 4	TB ≥ 17.40	0.950 (0.846–1.065)	TB ≥ 14.40	1.105 (0.383–3.188)
Model 2		0.949 (0.938–0.961)**		0.931 (0.896–0.967)**
Model 3		0.992 (0.960–1.024)		0.986 (0.909–1.069)
30 < age ≤ 60 Model 1				
TB Quartile 1	TB < 9.80 (μmol/L, reference)		TB < 8.00 (μmol/L, reference)	
TB Quartile 2	9.80 ≤ TB < 12.70	0.942 (0.834–1.065)	8.00 ≤ TB < 10.30	0.894 (0.709–1.128)
TB Quartile 3	12.70 ≤ TB < 16.40	0.924 (0.816–1.046)	10.30 ≤ TB < 13.30	0.856 (0.674–1.086)
TB Quartile 4	TB ≥ 16.40	0.940 (0.829–1.066)	TB ≥ 13.30	0.876 (0.679–1.131)
Model 2		0.980 (0.976–0.984)**		0.965 (0.958–0.973)**
Model 3		0.997 (0.989–1.006)		0.986 (0.965–1.006)
Age > 60 Model 1				
TB Quartile 1	TB < 10.20 (μmol/L, reference)		TB < 8.60 (μmol/L, reference)	
TB Quartile 2	10.20 ≤ TB < 13.00	1.075 (0.737–1.567)	8.60 ≤ TB < 10.70	1.046 (0.646–1.695)
TB Quartile 3	13.00 ≤ TB < 16.60	1.091 (0.741–1.607)	10.70 ≤ TB < 13.60	0.883 (0.544–1.434)
TB Quartile 4	TB ≥ 16.60	0.989 (0.669–1.461)	TB ≥ 13.60	1.139 (0.689–1.883)
Model 2		0.975 (0.964–0.985)**		1.000 (0.987–1.014)
Model 3		0.999 (0.973–1.026)		1.013 (0.974–1.054)

*TB* Total bilirubin, *OR* Odds ratio, *CI* Confidence interval

^Logistic regression model 1 with TB Quartile 1 as reference, including age, body mass index, blood pressure, alanine aminotransferase, blood urea nitrogen, creatinine, uric acid, total cholesterol, high-density lipoprotein, low-density lipoprotein and glucose as covariates

Model 2 and 3 analyzed TB as a continuous variable. Model 2 included TB as a covariate and model 3 included age, body mass index, blood pressure, alanine aminotransferase, blood urea nitrogen, creatinine, uric acid, total cholesterol, high-density lipoprotein, low-density lipoprotein and glucose as covariates

* P < 0.05, ** P < 0.01

## Discussion

Linear logistic regression analysis showed a negative correlation between TB and TG. Males showed significantly higher overall incidence of high TG than females. Men, who were younger than 60 years old, showed a higher incidence of high TG. For post-menopausal women aged more than 60 years, the incidence of high TG was higher. But binary logistic regression analysis indicated that TB could not be a risk factor for high TG. This means that there is no direct relationship between TG and TB.

At present, only a small number of studies refer to the relationship between TB and TG. And the recruitment volume of these studies is relatively small. There was no articles thought that TG was not related to TB. All of them found a negative relationship between TB and TG. For example, in a review which referred the relationship between TB and GS, Bulmer et al. [15] summarized that many study reported an inverse association between TB and TG. Lee et al. [13] said that TB was a protective factor for MS. At the same time, they found a negative relationship between TB and the development of high TG. But with a study of 6250 South Korean men, the results of this article had no gender universality. In the study cohort of Bhuiyan et al. [6], including 777 black and white subjects, TG were associated inversely with TB. The subjects were younger and less representative of the elderly. Besides, only few investigations studied the effect of sex and age on TB. To identifying the strongest predictors of TB levels, Topic et al. [21] investigated 97 males and 68 females who were aged from 39 to 65 years. In males, fasting serum insulin levels and fatty acids consumption were the strongest. In females, serum TG levels and fatty acids intake were the strongest. With a narrow age distribution and the small number of participants, it cannot represent the majority of the population. Madhavan et al. [20] have studied the age and gender distribution of TB in whites and blacks age from 5 to 30 years. In men, the level of TB increased with age up to 24 years. In women, it did not change significantly, except for preadolescent ages of 5–10 years. The negative association also existed between TB and TG in both genders. Further research was needed for people over 30 years of age. Oda et al. [9] have studied the cross-sectional and longitudinal relationship between TB and dislipidemia. They found that TB was significantly associated with prevalent high TG in men. In women, the association between them was marginal. However, the number of participants was too small (male: 2113, female: 1265) to find statistically significant. All the above articles showed a negative correlation between TB and TG. But none of them specifically investigate the relationship by further study.

The present study showed that there was no direct relationship between TB and TG in both genders. The negative correlation between them was disappeared when included other confounding factors. So it might imply that other confounding factors contributed to this relationship. Several reasons can be postulated. First, it was the role of oxidative stress. Heme oxidase catalyzes hemoglobin degradation to produce ferrous ions, carbon monoxide and biliverdin. The biliverdin reductase then reduces biliverdin to bilirubin [30]. Choi et al. [12] mentioned that MS always incorporated cardiovascular risk factors associated with insulin resistance. Both insulin resistance and impaired glucose tolerance were accompanied by oxidation. One feature of MS subjects was the elevated plasma levels of oxidized lipids. The other was that the antioxidant level was lower than normal. So they thought that TB levels were affected by insulin resistance-related dyslipidemia. This means that the negative correlation between TB and TG might be caused by oxidation. Bhuiyan et al. [6] also found that TB was negatively related to TG. They supposed that dislipidemia deplete the level of TB by promoting oxidative stress. Second, the negative relationship between TB and TG could be caused by estrogen. Recent researches showed that estrogen could protect women from cardiovascular diseases [31–33] by regulating liver lipid metabolism and serum lipoprotein levels [34]. Palmisano et al. [35–37] demonstrated that the ability of estrogen to reduce hepatic steatosis disappeared with the loss of Estrogen Receptor alpha (ERα) in a mouse model of hepatocyte ERα deficiency. It suggested that estrogen acted directly on the liver to reduce TG content through ERα. Hunsawong et al. [38] found that serum estrogen levels were significantly elevated in patients with cholangiocarcinoma and were positively correlated with TB. Kao et al. [39] reported that the latency of TB decline in women after liver transplantation was shorter than that in men, suggesting that E2/ERa signaling could reduce serum TB levels in regenerated liver. Therefore, it is possible that due to the action of estrogen on TG and TB, the two are negatively correlated with each other. Whatever the reason, there is no direct relationship between TB and TG. Of course, our research is by far the most detailed study of this relationship from the perspective of gender and age.

There are some limitations in this investigation. The present study was a cross-sectional study; it was unable to determine the causal relationship between TB and TG. The effects of direct and indirect bilirubin on TG need to be investigated. The blood indices were measured only once without repeated validations. Due to inadequate budget, inflammatory markers and sex hormones were not detected in the population. Serum TB might be influenced by genetic factors. The relationship between TB and TG may be more pronounced in people with diseases, for example in type II diabetes or obesity where high TG is present. The narrow range of TB and TG in healthy people makes it difficult to observe significant changes. Therefore, Future plans should require prospective and interventional investigations.

## Conclusions

In conclusion, our study showed no clear association between TG and TB in Chinese population. In most researches, the decline of TB was always accompanied by the increasing of TG. It’s hard to say that it wasn’t caused by other factors. The mechanism needed more prospective and interventional investigations to clarify.

## Abbreviations

ALT: Alanine aminotransferase
BH: Body height
BMI: Body mass index
BUN: Blood urea nitrogen
BW: Body weight
CBR: Conjugated bilirubin
Cr: Creatinine
DBP: Diastolic blood pressure
ERα: Estrogen Receptor alpha
G: Glucose
GS: Gilbert’s syndrome
HDL: High-density lipoprotein cholesterol
HO: Heme oxygenase
LDL: Low density lipoprotein cholesterol
MS: Metabolism syndrome
OR: Odd ratio
SBP: Systolic blood pressure
SD: Standard deviation
TB: Total bilirubin
TC: Total cholesterol
TG: Triglyceride
UA: Uric acid
